# Reconstructing the survival history of relict herbs endemic to the heavy snowfall regions of Japan during the Pleistocene

**DOI:** 10.1007/s10265-026-01727-y

**Published:** 2026-06-22

**Authors:** Watanabe Yoichi, Momoka Gozono, Hinata Koide, Hotaka Ishida, Koki Nagasawa, Kazutoshi Masuda, Shota Sakaguchi

**Affiliations:** 1 Graduate School of Horticulture, Chiba University, Matsudo 648, Matsudo, 271-8510 Chiba Japan; 2 Faculty of Horticulture, Chiba University, Matsudo 648, Matsudo, 271-8510 Chiba Japan; 3 Research Center of Genetic Resources, National Agriculture and Food Research Organization, Kan-nondai, Tsukuba, 3058517 Japan; 4 Graduate School of Human and Environmental Studies, Kyoto University, Yoshida-nihonmatsu-cho, Sakyo, 606-8501 Kyoto Japan; 5 Graduate School of Agriculture, Kyoto University, Kitashirakawa-oiwake- cho, Sakyo, 606-8502 Kyoto Japan

**Keywords:** Divergence time, Phylogeography, Plastome, Population genetic structure, Population size

## Abstract

The effects of past environmental changes on the demography and genetic diversity of relict species remain poorly understood. Such studies have rarely been conducted across multiple phylogenetically distant species, largely because of their rarity. Here, we investigated impacts of long-term climatic changes on the evolution and survival of three herbaceous Tertiary relict species endemic to heavy snowfall regions of Japan. Using population genomic analyses based on plastome sequences, we reconstructed phylogeographic patterns, including demographic histories, and explored potential drivers of similarities and differences among the species. Divergence times from close relatives were estimated at ca. 64 Ma for *Glaucidium palmatum*, 23 Ma for *Ranzania japonica*, and 12 Ma for *Paris japonica*, all of which predate the Pleistocene. The time to the most recent common ancestor (tMRCA) for each species ranged from approximately 0.03 to 3 Ma. Effective population sizes of species and regional groups expanded rapidly between approximately 100,000 and 10,000 years before present. Patterns of genetic diversity and geographic structure among the three relict species appear to reflect differences in dispersal ability and the locations of climatic refugia. Despite variation in tMRCAs among the three species, demographic expansions consistently occurred during the late Pleistocene and were likely associated with the development of heavy snowfall climate.

**Electronic supplementary material:**

The online version of this article (https://doi.org/10.1007/s10265-026-01727-y) contains supplementary material, which is available to authorized users.

## Introduction

Relicts represent distinctive species, or related species, that persist from a formerly larger and more widespread group that is now mostly extinct (Grandcolas et al. 2014). Since the Tertiary, as the global climate cooled and became drier, temperate plants in the Northern Hemisphere began to shift southward (Donoghue and Smith 2004). Climatic oscillations during the Pleistocene also had a significant impact on the temperate flora of the Holarctic (Momohara 2016). These changes led to the extinction of many temperate gymnosperm and angiosperm species that were unable to adapt to such climatic changes and/or survive as Tertiary relicts within climatic refugia (Milne and Abbott 2002; Tang et al. 2018). Elucidating the history of relicts that survived the Pleistocene can therefore help us to understand how past climate changes have shaped population dynamics and plant evolution. For example, genomic analyses of *Ginkgo biloba* (Ginkgoaceae), a well-known Tertiary relict gymnosperm, have revealed signatures of substantial demographic fluctuations, suggesting repeated range expansions and contractions during the Pleistocene glaciations, consistent with evidence from the fossil record (Hohmann et al. 2018; Zhao et al. 2019). Relicts can be categorized into two types—geographic and phylogenetic—based on their geographic distributions and phylogenetic relationships (Grandcolas et al. 2014). However, for Tertiary relicts, it is difficult to infer past geographic distribution for herbaceous species, which rarely leave fossil records. Nevertheless, defining relicts based on phylogenetic relationships remains feasible. In addition, demographic reconstructions based on genomic data can provide valuable insights into the evolutionary histories of relict plants.

East Asia is renowned for harboring numerous Tertiary relicts within the Holarctic flora (Tang et al. 2018). The Japanese archipelago, composed of continental islands, was shaped by the interplay of tectonic, geographic, and palaeoclimatic processes (Taira 2001). Its biota has been repeatedly fragmented by sea-level changes that intermittently separated the islands from the Eurasian continent. This allowed the continental islands to function as evolutionary cradles for neo-endemics or as refugia for Tertiary relict taxa that escaped harsh continental climates during the Pleistocene (Qian and Ricklefs 2000). Other examples of gymnosperm relicts in East Asia include the monotypic genus *Metasequoia* (Cupressaceae) in mainland China and *Sciadopitys* (Sciadopityaceae) in Japan, as well as angiosperm families such as Cercidiphyllaceae and Eupteleaceae which are endemic to East Asia, with some species restricted to Japan (Cao et al. 2020; Manchester et al. 2009; Zhu et al. 2020). Their continued survival in East Asia suggests the existence of a stable temperate climate with ample precipitation (Tang et al. 2018). Comparative population genomic analyses across multiple, phylogenetically distant species have revealed effects of long-term climatic changes on demography and genetic diversity (Milesi et al. 2024). However, due to their rarity and differences in habitat and distribution, the survival histories of relict species are often discussed in isolation, and the similarities and differences in their demography and genetic diversity remain insufficiently evaluated.

**Fig. 1:**
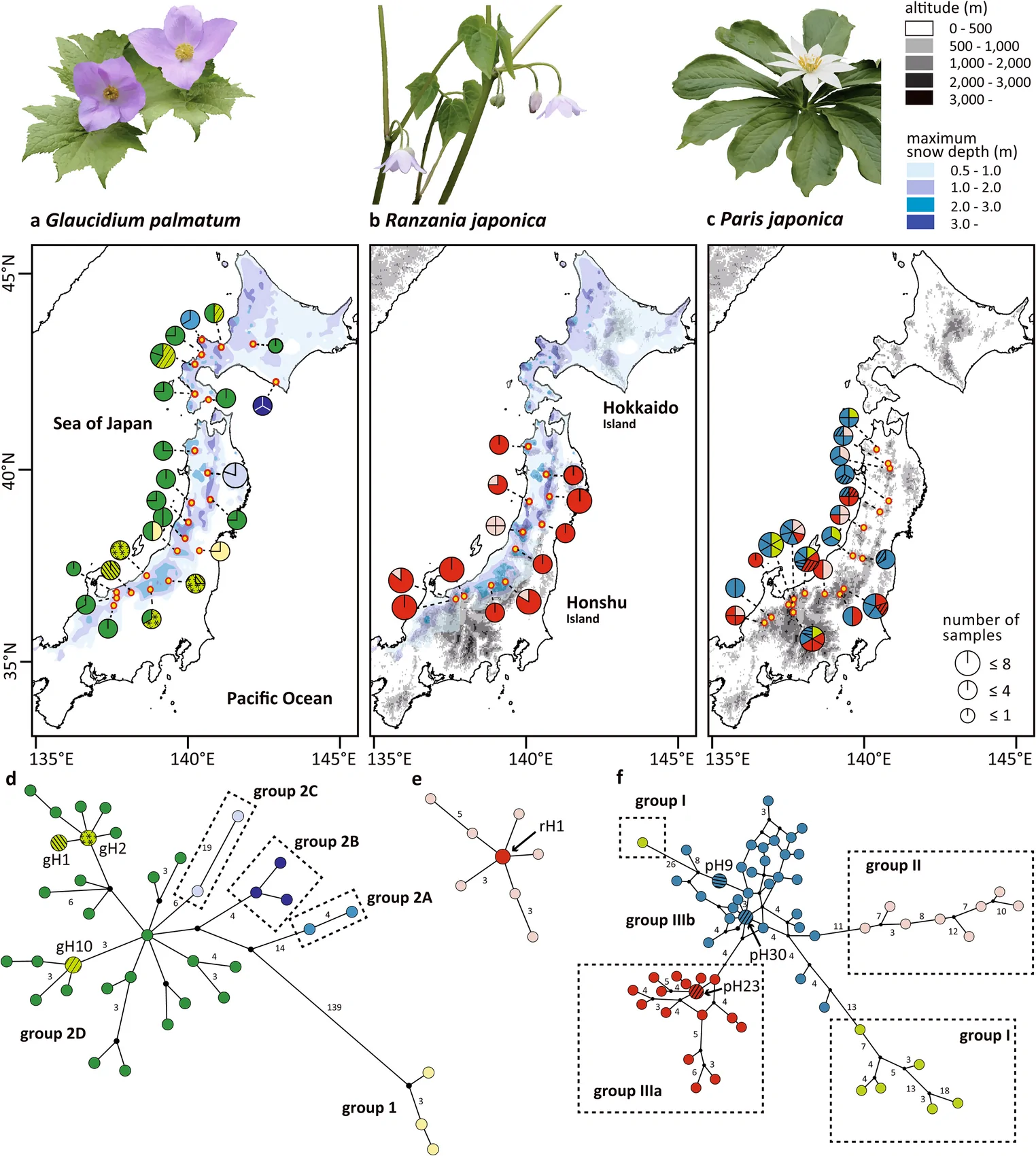
Distributions of the study populations and their haplotype networks, showing the haplo-groups and haplotypes of **a**,** d** *Glaucidium palmatum*, **b**,** e** *Ranzania japonica*, and **c**,** f** *Paris japonica*. In the distribution maps (a–c), the size of each pie chart reflects the number of samples. In the haplotype networks (d–f), haplotype codes are shown for those observed in more than one population, and the number of mutational steps (> 2) is shown above branches. The definition of groups is based on the results of a calibrated tree (Fig. 3), which was estimated with a Bayesian skyline

The snow depth in the Japanese archipelago, particularly along the Sea of Japan, is among the deepest in the temperate zone worldwide (Japan Meteorological Agency 2025; Kawase et al. 2018), with major mountain ranges covered by more than 200 cm of snow (Fig. 1). In this region, a unique type of vegetation is composed of plant species whose distributions closely correspond to those adapted to the heavy snowfall climate, known as the “Sea of Japan elements” (Fukuoka 1966; Maekawa 1949). This heavy snowfall is caused by three factors: the cold winter monsoon from the northwest, the warm Tsushima Current flowing into the Sea of Japan, and the continuous mountain ranges of Honshu and Hokkaido (Kawase et al. 2018). Clouds form when cold air masses encounter the warm current and are transported toward the Japanese mountains during strong winter monsoons, resulting in heavy snowfall. Heavy snowfall exerts diverse effects on temperate plant species in this area. The high snow pressure can damage the trunks and branches of woody species, favoring the survival and growth of species that are tolerant to this selective pressure (Homma 1997). Conversely, high humidity and groundwater derived from snowmelt can facilitate sprouting and subsequent plant growth. This characteristic is especially important for herbaceous plants, as there are species with larger bodies and leaves compared to those in light-snowfall areas along the Pacific Ocean, such as giant knotweed (*Fallopia sachalinensis*) in heavy snowfall and *F. japonica* in areas with lighter snowfall. For these reasons, the flora in this region exhibits distinct characteristics compared with that of regions experiencing light snowfall (Uemura 1989).

Remarkably, several relict plant species endemic to heavy snowfall regions have been identified. *Glaucidium palmatum* (Ranunculaceae), *Ranzania japonica* (Berberidaceae), and *Paris japonica* (Melanthiaceae) are characteristic relict species of the heavy snowfall regions. Morphological and phylogenetic evidence support the hypothesis that these three species represent phylogenetic relicts originating in the Tertiary period (Grandcolas et al. 2014; Ji et al. 2019; Sun et al. 2018; Zhai et al. 2019), although no fossils of these herbaceous species have been found. It is unclear how these relict species have survived past climatic changes. The timing of the development of the heavy snowfall climate remains controversial. Global cooling began in the mid-Miocene, and climatic oscillations started in the Pleistocene (Zachos et al. 2001). Eolian deposits in northern China suggest that the winter monsoon became progressively stronger during the Pleistocene (Sun et al. 2010), while the uplift of mountains in Honshu, Japan, began in the late Pliocene (Yonekura et al. 2001). In addition, paleovegetation reconstructions from middle Pliocene deposits in central Niigata, a heavy snowfall area (37.40–37.80°N, 138.46–138.50°E) along the Sea of Japan, suggest that this area had become colder and began experiencing heavy snowfall from this period (Momohara et al. 2017). It is unclear whether the snowfall climate was stable during the Pleistocene. During the last glacial period, which was the coldest period of the Pleistocene, it is estimated that snowfall decreased due to a decrease in inflow of the Tsushima current (Ono 1984). In addition, the paleovegetation from layers dating to the last glacial maximum (ca. 23,000 year BP) in northern Niigata, a heavy snowfall area (38.12°N, 138.29°E) along the Sea of Japan, includes *Abies homolepis*, *Picea jezoensis* var. *hondoensis*, and *Tsuga diversifolia*, species that are not typically associated with areas of heavy snowfall, suggesting that the snowfall may have been light in this period (Kamoi et al. 1988).

To evaluate how the development and persistence of heavy snowfall climates during the Pleistocene have influenced the evolution and survival of relict species, genetic analyses involving multiple species can provide valuable insights. We investigated the evolutionary processes and survival histories of three relict herbs using plastome sequences. Plastome analyses offer advantages of ease of sequencing and alignment, even among phylogenetically distant species, and facilitate haplotype determination in highly polyploid species (Uribe-Convers et al. 2014; Zhang et al. 2016). The uniparental inheritance of plastomes reflects specific modes of dispersal, most likely via seeds, which is essential for understanding the range expansion of plants (Duminil et al. 2007). We also evaluated the similarities and differences in the biogeographic patterns of Tertiary relicts in heavy snowfall regions by comparing the genetic diversity and demographic histories among the three species. Specifically, we aimed to address the following questions: (1) Did intraspecific divergence coincide with the development of heavy snowfall climates? (2) Were trends in population size change concordant among species and lineages? (3) Have the three relict species survived in geographically proximate areas? Our results provide a foundation for evaluating the effects of species on the past demography of relicts, as well as a better understanding of the evolutionary processes associated with Pleistocene climatic changes.

## Materials and methods

### Study species

The study species are *Glaucidium palmatum* Siebold et Zucc. (Ranunculaceae), *Ranzania japonica* (T.Itô ex Maxim.) T.Itô (Berberidaceae), and *Paris japonica* (Franch. et Sav.) Franch. (Melanthiaceae). The genus *Glaucidium* is monotypic and represents one of the earliest-diverging lineages in Ranunculaceae (Wang et al. 2009), with a divergence time estimated at ca. 89 Ma (Zhai et al. 2019). *G. palmatum* was once classified as the sole member of the monotypic family Glaucidiaceae (Hoot et al. 1999). This species is distributed from temperate to sub-boreal climates in the western part of Hokkaido and the northeastern part of Honshu (Fig. 1a). It grows widely in mountain forests and grasslands. Due to the presence of circular wings on its seeds, the seeds of *G. palmatum* appear to be dispersed by wind (Nakayama et al. 2010). The genus *Ranzania* is monotypic and is most closely related to the genus *Berberis*, which includes *Mahonia*, in Berberidaceae (Sun et al. 2018). The divergence time between *Ranzania* and *Berberis* is estimated as ca. 17 Ma (Sun et al. 2018). *R. japonica* is distributed in temperate zones in the northeastern part of Honshu (Fig. 1b). It grows in forests, especially on slopes along streams (Okawa and Sakata 2024). Because of its rarity, it is listed as Near Threatened (NT) in the Red List maintained by the Ministry of Environment of Japan (The Ministry of the Environment of Japan 2019). It produces white fleshy fruits containing seeds with elaiosomes, which are dispersed by birds and ants (Inoue 1994). The genus *Paris* (Melanthiaceae) comprises approximately 29 species and is widely distributed across Eurasia. The section *Kinugasa* is monotypic, including only *P. japonica*. Phylogenetic studies revealed that this section represents an ancestral lineage that contributed to the establishment of species in other sections of the genus *Paris*, which includes most species, through ancient hybridization with the sect. *Paris* (Zhou et al. 2024). A previous phylogenetic study has indicated that *P. japonica* diverged from its congeners ca. 16 Ma (Ji et al. 2019). Notably, *P. japonica* is an octoploid species with a chromosome number of 2n = 8x = 40 (Haga 1937), and possesses the second-largest known eukaryotic genome (148.89Gb; Fernández et al. 2024). It is distributed in the sub-boreal climatic zone in the northeastern part of Honshu (Fig. 1c). It grows in open habitats such as forest edges. Its seeds are dispersed by at least two mammals, *Vulpes vulpes japonica* and *Martes melampus* (Ueuma et al. 2005). Based on field observations and flower morphology, all three species flower in early spring, and honeybees and bumblebees are likely to be effective pollinators for all the study species (Inoue 1994).

## Plant sampling and DNA extraction

We collected leaves from 79 samples of *G. palmatum* across 23 localities, 50 samples of *R. japonica* from 12 localities, and 70 samples of *P. japonica* from 18 localities, and immediately dried them using silica gel (Fig. 1, Table S1). Genomic DNA was extracted from the dried leaves using the modified CTAB method (Murray and Thompson 1980) or the DNeasy Plant Mini Kit (Qiagen, Hilden, Germany).

## Plastome sequencing

The plastome was amplified by long-range polymerase chain reaction (PCR) using angiosperm universal primers (Uribe-Convers et al. 2014; Zhang et al. 2016). Different primer pairs were used for each species (Table S2). PCR was carried out in a 10 µL volume, containing 0.2 µL of PrimeSTAR GXL DNA polymerase (Takara Bio, Otsu, Japan), 2.0 µL of 5× PrimeStar GXL buffer, 0.8 µL of dNTP mixture, 0.2 µL of each primer (10 µM) and 1.0 µL of template DNA (containing 1.0–10 ng of genomic DNA). The PCR was performed with 30 cycles of 10 s at 98 °C and 10 min at 65 °C, with a final extension of 10 min at 75 °C. After purifying the amplicons with AMPure XP (Beckman Coulter, Indianapolis, IN, USA), amplicons from each primer pair of a given sample were pooled to a final concentration of 0.2 ng/µL and used to construct a sequencing library using an Illumina Nextera XT Library Preparation Kit (Illumina, San Diego, CA, USA), following the manufacturer’s instructions. Libraries were sequenced with 150 bp paired-end reads on two lanes of the HiSeq X platform (Illumina, San Diego, CA, USA).

## Calling of single nucleotide polymorphisms

Low-quality reads and adapter sequences were filtered from the raw reads using fastp v.0.20.1 (Chen et al. 2018). Clean reads were mapped to the respective plastome references for *G. palmatum* (Zhai et al. 2019; accession number MK569492; 156,791 bp), *R. japonica* (Sun et al. 2018; MH423072; 169,224 bp) and *P. japonica* (Yang et al. 2019; MH796668; 155,957 bp), using the BWA-MEM algorithm implemented in bwa v.0.7.17 (Li 2013) with default settings. One of the inverted repeat regions was removed from the genome references before read mapping. Additionally, complete plastome sequences of seven or eight outgroup species for each family were chosen from the NCBI database (Table S3), and mapped onto the study species reference for phylogenetic reconstruction. Because *G. palmatum* represents the earliest-diverging lineage within Ranunculaceae, *R. japonica* of Berberidaceae, which is the most closely related family to Ranunculaceae, was used as one of the outgroups. As raw reads were not available for the outgroup species; therefore, complete plastome sequences for the outgroups were mapped to each species’ reference after random fragmentation using GemSIM v.1.6 (McElroy et al. 2012). After sorting the mapped reads using samtools v.1.11 (Danecek et al. 2021), PCR duplicates were removed using MarkDuplicates implemented in GATK v.4.1.9.0 (McKenna et al. 2010). Variant sites (single nucleotide polymorphisms, SNPs) were called using FreeBayes v.0.9.21 (Garrison and Marth 2012) with the setting --ploidy 1, and filtered using vcftools v.0.1.16 (Danecek et al. 2011) with the following settings: DP > 10 (exceeding 10 reads at a site for each individual), -max-alleles 2, --min-alleles 2 (using only bi-allelic sites), --minQ 40, --min-meanDP 20, --max-missing 1.0 (no missing allowed). Invariant sites were called using HaplotypeCaller implemented in GATK with the settings -ERC GVCF and -ploidy 1; genotyped using CombineGVCFs and GenotypeGVCFs with the setting -all-sites; extracted using SelectVariants with the setting --select-type-to-include NO_VARIATION; and filtered using vcftools with the settings DP > 10 and --max-missing 1.0. Variant and invariant sites were combined for the calculation of nucleotide diversity and demographic analyses. Haplotypes were identified separately using DnaSP v.6 (Rozas et al. 2017) for data with and without outgroups.

## Phylogenetic relationships and divergence time estimation with outgroups

Phylogenetic relationships including outgroups for each study species were inferred based on variant sites. Maximum likelihood trees were constructed using RAxML v. 8.2.0 (Stamatakis 2014). In the analysis, a GTR + Γ substitution model was used, and node supports were assessed by bootstrap analysis with 1,000 replicates. To estimate the time to the most recent common ancestor (tMRCA) for each study species, a Bayesian phylogenetic approach was used to construct a time-calibrated tree using BEAST v.2.6.6 (Bouckaert et al. 2019). To equalize the number of samples from each species, only two haplotypes per study species, which were the most diverged within each species based on the maximum likelihood tree, were used for age estimates. A GTR + Γ + I substitution model, a relaxed clock using a log-normal distribution and a Yule speciation process were used as tree priors, which were justified by Bayes factors. Each tree was calibrated in three nodes with uniform priors using the 95% highest posterior densities (95%HPDs) from the angiosperm tree, calibrated with fossil records (Magallón et al. 2015) for Ranunculaceae and Berberidaceae. A tree for Melanthiaceae was calibrated using the monocots tree (Givnish et al. 2016), because the tree by Magallón et al. (2015) is not well-suited for Melanthiaceae. Calibrated nodes for Ranunculaceae were applied as follows: Berberidaceae and Ranunculaceae, 68.17–95.84 Ma; Ranunculaceae, 58.98–79.51 Ma; Ranunculaceae excluding *G. palmatum*, 55.87–70.00 Ma. Calibrated nodes for Berberidaceae were applied as follows: Berberidaceae, 33.97–72.41 Ma; the clade including *Podophyllum*, *Caulophyllum* and *Nandia*, 26.23–67.46 Ma; the clade including *Caulophyllum* and *Nandina*, 16.59–57.28 Ma. Calibrated nodes for Melanthiaceae were applied as follows: Melanthiaceae, 61.9–106.9 Ma; the clade excluding *Veratrum patulum*, 50–97 Ma; the clade including *Paris*, *Trillium* and *Xerophyllum*, 30–80 Ma. The default settings in BEAUti v.2.6.6 were used for all other priors. The run consisted of 200 million Markov chain Monte Carlo (MCMC) steps, and the parameters were sampled every 50,000 steps. Stationarity and convergence were assessed after discarding the first 20 million steps as burn-in, based on effective sample size (ESS > 500) using Tracer v.1.5. A consensus tree with mean divergence times and 95% HPD intervals on nodes was exported using TreeAnnotator.

### Phylogenetic relationships and ancestral area reconstruction using time-calibrated tree without outgroups

Haplotype networks were constructed for each species by the median-joining method using PopART v.1.7 (Leigh and Bryant 2015). Phylogenetic relationships and divergence times among haplotypes were estimated using BEAST. For age estimation, both variant and invariant sites were analyzed by applying a coalescent approach with a known substitution rate of chloroplast DNA (Muse 2000; Wolfe et al. 1987). A GTR + Γ + I substitution model, a strict molecular clock and a coalescent Bayesian Skyline model were used as priors (Drummond et al. 2005). The time-calibrated tree based on fossils is highly dependent on the number of calibration points, assignment accuracy, and so on (Drummond and Rambaut 2007). Therefore, we used the known mutation rate to scale the tree of each study species, in order to compare it with the uncertainty of the calibrated trees analyzed in the previous section. Absolute time was scaled by adopting the substitution rate of 4.0 × 10^− 10^ per site per year, derived from the moderate substitution rate of 2.0 × 10^− 9^ (Muse 2000; Wolfe et al. 1987), divided by an assumed generation time of five years for each species. This approach was adopted, because per-year substitution rates of perennial plants are influenced by their generation times (*R*. *japonica*, Sanyasou-Tsurezurenikki 2015; *P*. *japonica*, Shioukan 2024; *G. palmatum*, Shuminoengei 2014). The upper limit of population size was set to 100 million. The default settings in BEAUti were used for all other priors. The run consisted of 100 million MCMC steps, and the parameters were sampled every 10,000 steps. The stationarity and convergence of the estimations after a burn-in period of 50 million steps were assessed using Tracer.

The ancestral areas and biogeographic events for each study species were estimated by statistical dispersal–vicariance analysis (S-DIVA) implemented in RASP v.3.2 (Yu et al. 2020). The time-calibrated tree was used and defined three geographic areas for estimating the ancestral area, i.e., A, Hokkaido; B, northern Honshu; C, central Honshu. This definition was expected to detect effects of insular isolation between the two islands and montane isolation between the northern and central regions of Honshu, as observed in alpine and snow-dependent plants (Ikeda 2022; Masuda et al. 2024). To account for phylogenetic uncertainty, we used 50,000 randomly selected trees from the BEAST output data for the S-DIVA analysis. The analysis was performed using default settings, and the maximum number of areas for all nodes was set to two.

## Genetic diversity and historical population size change

Nucleotide diversity (Nei 1987) and haplotype richness within populations, after rarefaction with two individuals, were calculated for populations, geographic regions, and the total population using DnaSP and Contrib v.1.02 (Petit et al. 1998), respectively. Genetic differentiation estimate, *F*_ST_, was calculated between populations (Hudson et al. 1992) using DnaSP. Because genetic divergence between groups 1 and 2 of *G. palmatum* was pronounced, indicating substantial heterogeneity between the groups, and because haplotypes of group 1 were found only from two populations, the above parameters were calculated exclusively for populations containing haplotypes derived from group 2. Tajima’s *D* (Tajima 1989a, b a) and Fu’s *Fs* (Fu 1997), as estimates for changes in population size (Tajima 1989a, b b), were calculated within phylogenetic groups for each species, which were defined based on the time-calibrated tree, using Arlequin v.3.5 (Excoffier et al. 2005). Mismatch distribution analyses (Rogers and Harpending 1992) were conducted within species and phylogenetic groups by estimating parameters under a sudden population expansion model using Arlequin. The sum of squared deviations between the observed and expected distributions (SSD, Rogers and Harpending 1992) and the raggedness index of the observed distribution (HRI, Harpending 1994) were used as test statistics to assess the goodness of fit.

Historical changes in effective population size for each species were reconstructed using the Bayesian skyline method implemented in BEAST (Drummond et al. 2005). This calculation was conducted simultaneously with the estimation of the phylogenetic tree without outgroups, as described in the previous section. In addition, a Bayesian skyline for each phylogenetic group, defined based on the calibrated tree for each species, was estimated with the same prior settings as those used above. Bayesian skyline analysis was not performed for group 1 of *G. palmatum* because there were only three haplotypes in this group. Estimates of effective population sizes through time were obtained after discarding a burn-in of 10 million MCMC steps using Tracer.

## Results

### Polymorphisms

Two lanes of Illumina sequencing yielded 2.1–9.9 million paired-end reads per sample, with an average of 4.9 million. The average depth of *G. palmatum*, *R. japonica*, and *P. japonica* was 2845.9, 1737.8, and 1174.0, respectively. The number of detected SNPs ranged from 286 to 2,344 with outgroups, and from 15 to 244 without outgroups (Table S4). The numbers of haplotypes detected in the *G. palmatum*, *R. japonica*, and *P. japonica* datasets with outgroups were 23, 8, and 53, respectively, whereas those without outgroups were 37, 8, and 62, respectively. The total number of sites detected ranged from 56,918 to 66,363 bp with outgroups, and from 84,388 to 115,061 bp without outgroups (Table S4).

### Phylogenetic relationships and evolutionary time with outgroups

Phylogenetic relationships with outgroups, based on the maximum likelihood method, indicated each study species was monophyletic (Fig. S1). The divergence times from the closest relatives of *G. palmatum*, *R. japonica*, and *P. japonica* were estimated as 63.66 Ma (95% HPD: 58.98–71.27 Ma), 23.28 Ma (95% HPD: 9.49–40.01 Ma), and 11.58 Ma (95% HPD: 5.10–22.56 Ma), respectively (Fig. S2). The times to the most recent common ancestor (tMRCAs) of *G. palmatum*, *R. japonica*, and *P. japonica* were estimated as 3.52 Ma (95% HPD: 1.43–8.23 Ma), 0.77 Ma (95% HPD: 0.06–2.65 Ma), and 1.32 Ma (95% HPD: 0.26–3.33 Ma), respectively.

### Phylogenetic relationships and distribution of haplotypes (or haplogroups) and their historical changes

**Fig. 2:**
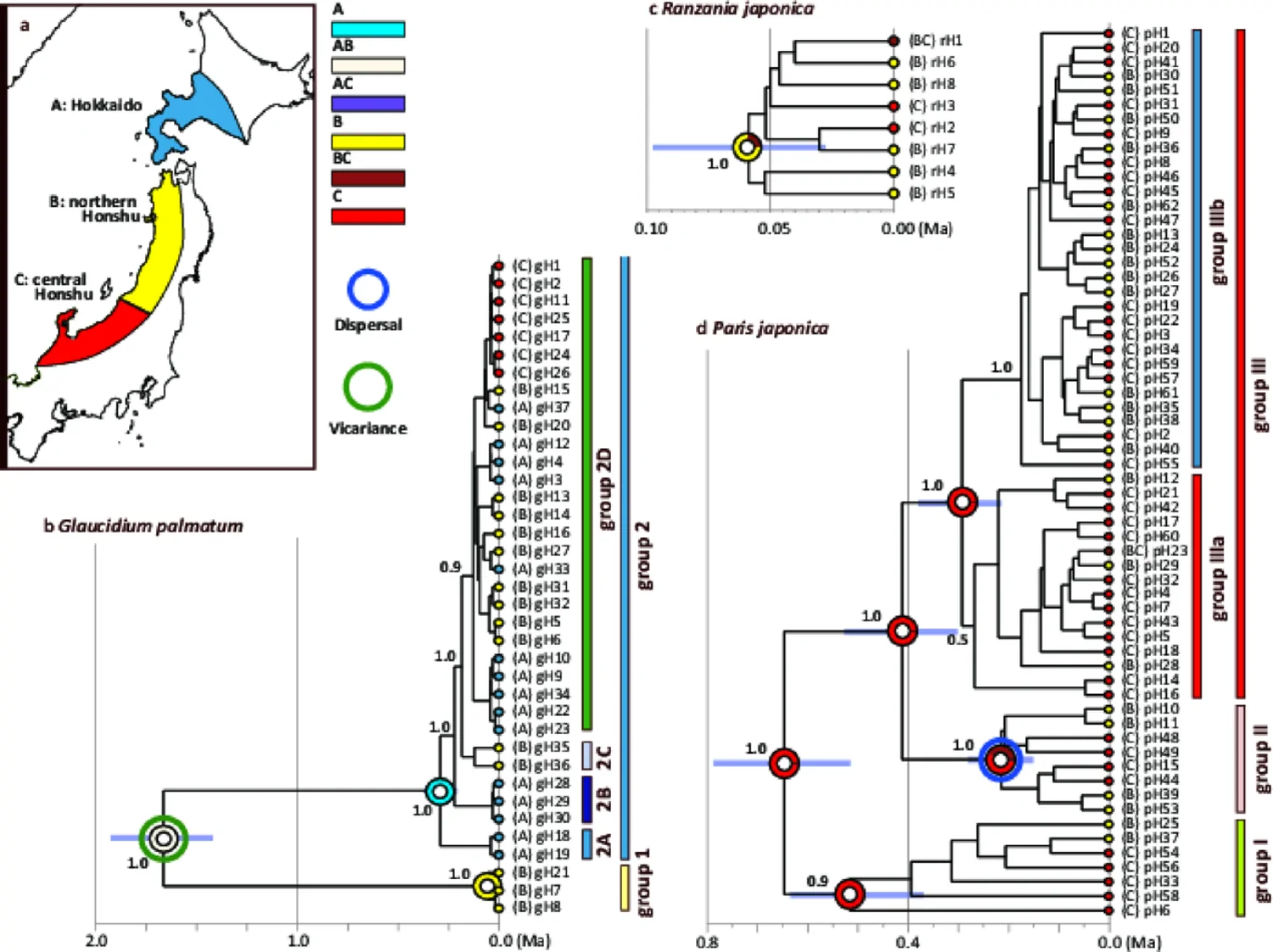
Ancestral area reconstruction based on a calibrated tree, estimated with a Bayesian skyline, for each species. **a** The map of defined three areas, and result of reconstruction for **b** *G. palmatum*, **c** *R. japonica*, and **d** *P. japonica*. Bars show the 95% highest posterior density (95%HPD) of divergence time for the main clades with a high posterior probability (PP > 1.0). Posterior probabilities are shown above nodes. The node for the most recent common ancestor of each species was calibrated. A haplotype code and its present distribution are indicated at the tip of a branch. The pie charts at internal nodes represent the marginal probabilities for each possible ancestral area. The map represents the geographic areas used for the ancestral area reconstruction

Phylogenetic analyses without outgroups estimated the tMRCAs for *G. palmatum*, *R. japonica*, and *P. japonica* as 1.66 Ma (95% HPD: 1.42–1.92 Ma), 0.06 Ma (95% HPD: 0.03–0.10 Ma), and 0.64 Ma (95% HPD: 0.52–0.79 Ma), respectively (Fig. 2). We identified distinct phylogeographic patterns in haplotype distributions between *G. palmatum* and the other species. In *G. palmatum*, we identified two groups (groups 1 and 2) with the highest posterior probability (PP = 1.0; Fig. 2a), and these groups were separated by 139 steps in the median-joining network (Fig. 1d). The three haplotypes of group 1 were found only in two populations located in northern Honshu. When group 2 was subdivided into a monophyletic group 2B and a paraphyletic group 2A, the haplotypes of group 2A were mainly distributed in more northerly regions, especially in Hokkaido (Fig. 1a). The 27 haplotypes of group 2B were observed across the entire species’ range, and all the haplotypes from central Honshu formed a monophyletic group within group 2B (Fig. 2a). In contrast, no clear phylogenetic groups were identified in *R. japonica* (Fig. 2b). One haplotype, rH1, was found in 88% of the analyzed samples. Each of the other haplotypes was identified from a single sample, and four out of seven haplotypes were found in one population located in northern Honshu (Fig. 1b). In *P. japonica*, we identified three groups (groups I–III) with high probabilities (PP > 0.9), with monophyletic group IIIb and paraphyletic group IIIa in group III (Fig. 2c). Only three of the 62 haplotypes were shared among populations, and 58 of the 62 were singleton haplotypes (Tables S1, S4). We did not find any clear spatial patterns in the distribution of haplogroups (Fig. 1c).

Ancestral area reconstruction provided the highest probability for the ancestral area of the MRCA of *G. palmatum*, *R. japonica*, and *P. japonica* to be AB (100%), B (99%), and C (76%), respectively. In addition, a vicariance event was inferred for the split between groups 1 and 2 for *G. palmatum* (Fig. 2a).

### Genetic diversity and historical population size change

**Fig. 3:**
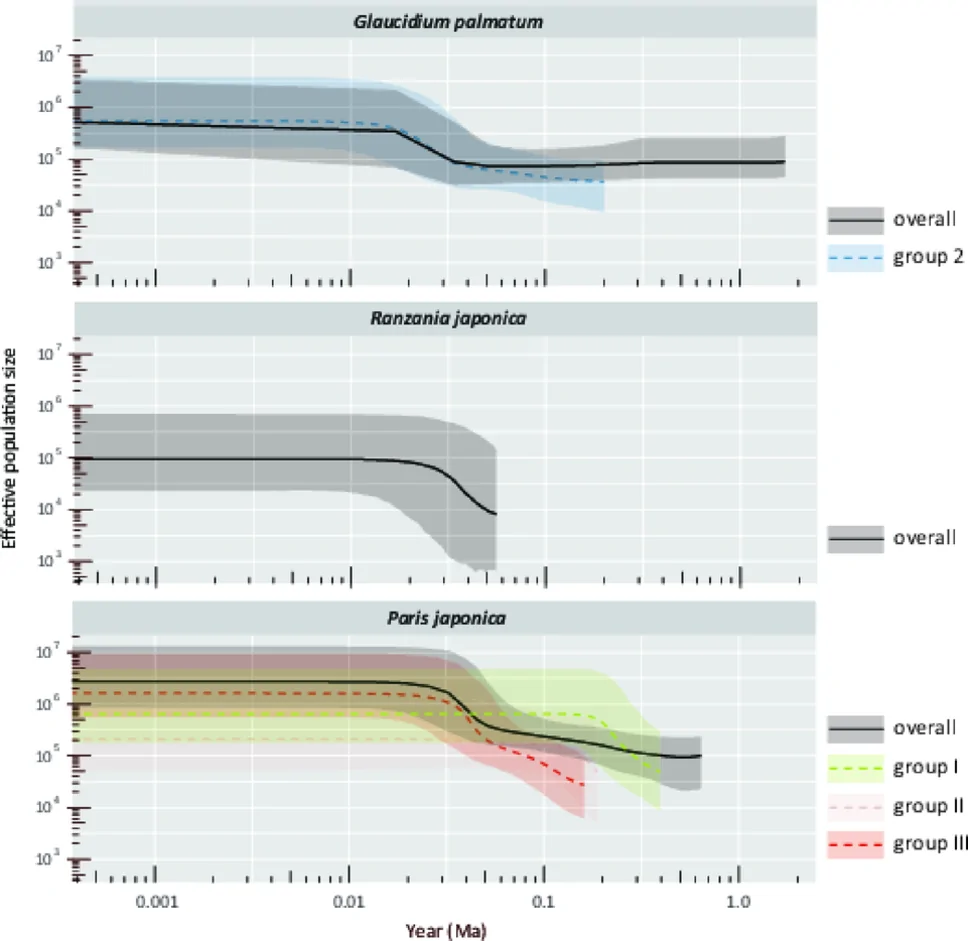
Population size changes for each species and phylogenetic group reconstructed with a Bayesian skyline plot, with an assumed generation time of five years. Lines and shaded areas indicate posterior nodes and 95% highest posterior densities (95%HPD), respectively

The nucleotide diversity in the total population of *G. palmatum*, *R. japonica*, and *P. japonica* was 0.00022, 0.00001, and 0.00021, respectively, and genetic differentiation among populations was 0.63, -0.01, and 0.15, respectively (Table 1). When genetic diversity indices were calculated for the three geographic regions as defined in Fig. 3, the nucleotide diversity of *G. palmatum* was highest in northern Honshu (0.00043), due to the presence of group I. In contrast, no clear geographic pattern was detected in *R. japonica* with low polymorphism within the species (Table 1).

**Table 1:** Polymorphism and genetic diversity estimates for *G. palmatum*, *R. japonica* and *P. japonica*

Species	Region	*N*	*p*	h	Pi	(SE)	HR_[2]_	(SE)	F_ST_
*Glaucidium palmatum*									
	overall	79	23	37	0.00022	0.00001	0.45	0.07	0.71
	Hokkaido	28	8	15	0.00009	0.00000	0.57	0.11	–
	northern Honshu	29	8	15	0.00043	0.00002	0.56	0.11	–
	central Honshu	22	7	7	0.00001	0.00000	0.31	0.14	–
	group 2	73	21	33	0.00007	0.00000	0.42	0.05	0.84
*Ranzania japonica*									
	overall	50	12	8	0.00001	0.00000	0.18	0.09	-0.01
	northern Honshu	22	7	6	0.00001	0.00000	0.21	0.15	–
	central Honshu	28	5	3	0.00000	0.00000	0.12	0.08	–
*Paris japonica*									
	overall	70	18	62	0.00021	0.00002	0.98	0.06	0.15
	northern Honshu	28	8	24	0.00018	0.00001	0.88	0.09	–
	central Honshu	42	10	39	0.00022	0.00000	0.99	0.01	–

*N*, number of samples; *p*, number of populations; *h*, number of haplotypes; *Pi*, nucleotide diversity with standard error (SE); *HR*_[2]_, average haplotype richness of populations after applying a rarefaction for two individuals, with standard error (SE); *F*_ST_, genetic differentiation among populations (Hudson et al. 1992)

The two neutrality statistics, Tajima’s *D* and Fu’s *Fs*, which are used as estimates of changes in population size, were significantly negative, suggesting demographic expansion for the total populations of *G. palmatum* and *R. japonica*, and Fu’s *Fs* was significantly negative for *P. japonica* (Table 2). Of the phylogenetic groups, groups 1 and 2 of *G. palmatum* and group III of *P. japonica* were significantly negative for both statistics. The SSD and HRI indices for the mismatch distribution analyses indicated that the observed distributions of each species and phylogenetic group fitted to a sudden population expansion model (Table 2). Excluding group 1 for *G. palmatum* and groups I and II for *P. japonica*, the mismatch distribution for each species and group had a clear unimodal distribution (Fig. S3). In the Bayesian skyline plot, population expansions for the three species, including group 2 for *G. palmatum* and group III for *P. japonica*, were observed in a recent timeframe of around 0.01–0.1 Ma (Fig. 3).

**Table 2:** Neutrality tests and mismatch distribution analyses for *G. palmatum*, *R. japonica* and *P. japonica*

Species	Group	*N*	h	θ	D		Fs		SSD		HRI	
*Glaucidium palmatum*												
	overall	79	37	4.30E–4	−1.690	^*^	−24.02	^***^	0.008	^NS^	0.007	^NS^
	group 1	5	3		−1.124	^NS^	−3.07	^**^	0.093	^NS^	0.230	^NS^
	group 2	74	34		−2.068	^***^	−24.74	^***^	0.005	^NS^	0.008	^NS^
*Ranzania japonica*												
	overall	50	8	2.95E–5	−2.412	^**^	−3.40 E + 38	^***^	0.010	^NS^	0.407	^NS^
*Paris japonica*												
	overall	70	62	2.80E–4	−0.892	^NS^	−24.13	^***^	0.001	^NS^	0.003	^NS^
	group I	7	7		−0.204	^NS^	0.04	^NS^	0.037	^NS^	0.073	^NS^
	group II	8	8		−0.048	^NS^	−1.50	^NS^	0.031	^NS^	0.082	^NS^
	group III	55	47		−1.377	^NS^	−21.73	^***^	0.003	^NS^	0.008	^NS^

*N*, number of samples; *h*, number of haplotypes; *θ*, Watterson’s *θ*; *D*, Tajima’s *D* (Tajima 1989a, b); *Fs*, Fu’s *Fs* (Fu 1997); SSD, the sum of square deviations between the observed and expected distributions (Rogers and Harpending 1992); HRI, the raggedness index of the observed distribution (Harpending 1994)

^NS^, not significant; *, *p* < 0.05; **, *p* < 0.01; ***, *p* < 0.001

## Discussion

### When did the relict species diverge from extant relatives and start to diverge within species?

The plastome data support the hypothesis that *G. palmatum*, *R. japonica*, and *P. japonica* are phylogenetic relicts, dating back at least to the Tertiary. The divergence times of focal species from their close relatives predate the Pleistocene (Fig. S2), and are consistent with previous phylogenetic studies for each family with sufficient taxa (Sun et al. 2018; Yang et al. 2019; Zhai et al. 2019).

In contrast, the tMRCA for *G. palmatum*, *R. japonica*, and *P. japonica* ranged from approximately 0.03 to 3 Ma (Fig. 2), suggesting that each species began to diversify during the late Pliocene to Pleistocene. The tMRCA for each species is debatable because only a few old calibration points were used in the age estimates with outgroups (Fig. S2). However, the estimation errors for tMRCAs based on the two methods (i.e., calibration-based and substitution rate-based) overlapped, indicating the tMRCA estimates are not in major conflict. One hypothesis regarding the survival of relicts, such as those from the Tertiary, is that they have limited ability to shift their distribution ranges as the climate changes and remain in limited habitats, or refugia, undergoing numerous extinction events (Tang et al. 2018; Zhao et al. 2019). In the case of relicts observed today in the heavy snowfall climate of the Japanese archipelago, it is hypothesized that the ancestral lineages once grew in the cold and snowy climates that existed in high-latitude areas of the Asian continent during the Miocene, and subsequently colonized and remained in the southerly snowy areas that arose as a result of climatic changes during the Pleistocene (Milne 2006; Milne and Abbott 2002). Therefore, the tMRCA for each species is expected to be related to the development of a heavy snowfall climate or the colonization period in the Japanese archipelago, as migration routes into the islands are limited. Fossil and phylogenetic analyses suggest that the distributions of temperate relict gymnosperms shrank in response to the cold and arid climate that expanded globally during the Pleistocene (Lin et al. 2022), and they have survived in suitable habitats in East Asia through niche conservatism (Tang et al. 2017, 2018). In Japan, the extinction of living fossils such as *G. biloba* and *M. glyptostroboides* during the late Miocene to early Pleistocene indicates that a cold and arid climate affected Japanese flora (Momohara 1994; Momohara et al. 2017). Palaeobotanical and palaeoclimatic evidence suggests that the heavy snowfall climate arose after 1.5–1.8 Ma (Momohara et al. 2017; Yonekura et al. 2001; Zachos et al. 2001), but direct botanical evidence remains limited. The tMRCAs for the three species may therefore provide additional evidence for the development of a heavy snowfall climate (Yoichi et al. 2023).

The variations in tMRCAs and ancestral areas among the MRCAs of the three species (Fig. 2) may reflect different responses to climatic changes. Ancestral area reconstruction helps to infer the survival areas of the three relict species before their range expansions. The different ancestral areas suggested for the three species—i.e., Hokkaido and northern Honshu for *G. palmatum*, northern Honshu for *R. japonica*, and central Honshu for *P. japonica* —indicate that their MRCAs expanded from refugia in different regions at different times. The recent intraspecific divergence of *R. japonica* during the late Pleistocene is concordant with the survival history of *G. biloba* (Hohmann et al. 2018), suggesting significant extinction events during the climatic oscillations, which could have extirpated the genetic variation in modern populations. In contrast, the oldest tMRCA of *G. palmatum*, based on the two methods used (1.43–8.23 Ma and 1.42–1.92 Ma) supports long-term persistence, as for other relict genera in the temperate flora of East Asia, such as *Cercidiphyllum* and *Euptelea* (Cao et al. 2020; Zhu et al. 2020).

### Demographic history of species and lineages

Although the tMRCAs of the three species were different, we found a similarity in their demographic histories. A rapid demographic expansion was detected in *R. japonica* (Fig. 3). The most recent tMRCA of *R. japonica*, based on the two methods used (0.06–2.65 Ma and 0.03–0.10 Ma), was associated with the lowest nucleotide diversity and present effective population size, suggesting a higher vulnerability to the climatic oscillations compared with the other two species. The negative Tajima’s *D* value (−2.412), which deviated significantly from zero, suggests a rapid range expansion from small refugia. Such demographic expansion was observed in the total population of each species, as well as in group 2 of *G. palmatum* and group III of *P. japonica* (Fig. 3). The effective population sizes of the species and groups expanded rapidly between approximately 100,000 and 10,000 years ago. The paleovegetation study by Kamoi et al. (1988) suggested that the extent of the heavy snowfall climate may have decreased during the last glacial maximum. This evidence suggests that the area of heavy snowfall climate, which is suitable for their survival, may have changed during this period (EPICA community members 2004).

Meanwhile, the three relict species also exhibited differences in their demographic histories, particularly *G. palmatum*, which can be distinguished into two distinct lineages. The isolated distribution of group 1 in the central species range indicates that the group is highly relictual. In addition, because the population of *G. palmatum*, which predominantly harbored haplotypes of group 1, is exceptionally found in a light snowfall area (Figs. 1 and 2), the survival of group 1 may not be related to the spatial expansion of a heavy snowfall climate. In contrast, the widespread distribution of group 2 suggests that the lineage would be adaptive to a heavy snowfall climate. The presence of two diverged groups, which appear to occupy distinct ecological niches, suggests that adaptation to heavy snowfall environments may have involved a niche shift during the late Pleistocene. Although niche conservatism is often reported in relict species (Wiens and Graham 2005), empirical demonstrations of niche shifts remain limited (Worth et al. 2014). Therefore, careful discussion is needed when interpreting signals of demographic expansion, as such expansions may have occurred after ecological niche shifts. The long branch length between the two groups indicates significant extinction events followed by a demographic expansion, as suggested by Tajima’s *D* and Fu’s *Fs* values, mismatch distribution analysis, and the Bayesian skyline plot. In *P. japonica*, the distribution of nucleotide differences between individuals for groups I and II was not unimodal, which does not support a sudden population expansion (Fig. S3), although the other two groups of *P. japonica* suggested a sudden population expansion model in the mismatch distribution analysis (Table 2). This result suggests that groups I and II of *P. japonica* have maintained stable population sizes (Figs. 3, S3). This species grows in higher altitudinal areas than the other two species, where sub-boreal forests are established. It is known that sub-boreal forests dominated in central and northern Honshu during glacial periods (Tsukada 1985). Therefore, this species, which is particularly adapted to cold climates, may have maintained its stable population size during the late Pleistocene.

### Range shifts during the late Pleistocene

The distributions of haplotypes, including rare haplotypes, suggest migration and stability for each species and region. In particular, the genetic structure based on variation in the plastome, with uniparental inheritance, reflects different modes of dispersal, most likely via seeds (Duminil et al. 2007; Kuroiwa 2010). *G. palmatum* exhibited a clear genetic structure, showing the highest genetic differentiation among populations (*F*_ST_ = 0.71). The lineage of group 1 is unique, which may have led to the highest *F*_ST_. However, even when calculating *F*_ST_ without the two populations including group 1, *F*_ST_ was still high at 0.84, suggesting that long-distance dispersal in this species may be limited (Nakayama et al. 2010). Twelve of the 23 haplotypes, which were endemic to Hokkaido (Fig. 2a), suggest long-term persistence on the island, while low nucleotide diversity (0.00001) in central Honshu suggests a recent southward range expansion (Fig. 2a). This is not concordant with the paradigm of a northward expansion from southern glacial refugia in the Northern Hemisphere (Hewitt 2000), as seen in the snow-dependent herb *Arnica mallotopus* (Masuda et al. 2024) and the heavy snowfall ecotype of the temperate conifer *Cryptomeria japonica* (Kimura et al. 2014). Because this theory has been established primarily based on temperature fluctuations during the climatic oscillations, this discordance may indicate that the survival of plants that are endemic to heavy snowfall climate depends more on spatiotemporal changes in snow cover than on temperature. Kimura et al. (2014) and Masuda et al. (2024) have examined the habitat connectivity of populations using distribution modeling; in particular Masuda et al. (2024) studied the influence of spatiotemporal changes in snow cover on habitat connectivity and suitability. Although the spatial pattern of genetic diversity in *G. palmatum* is not the same as that of *A. mallotopus*, *G. palmatum* populations may have also been influenced by spatiotemporal changes in suitable habitats associated with snowfall.

In contrast, clear spatial genetic patterns were not observed in the other two species. *R. japonica*, in particular, exhibited a weakly defined genetic structure, with the lowest genetic differentiation among the populations, which is consistent with a recent range expansion from bottlenecked refugia. *P. japonica* also exhibited a weakly defined genetic structure, possessing the highest number of haplotypes within the total population (*h* = 62) and the highest haplotype richness within the regions (*HR* = 0.88–0.99) among the three species. This suggests that frequent dispersal among stable refugia has resulted in a weakly defined genetic structure characterized by high genetic diversity.

## Conclusions

The approach using the plastome seems suitable for comparative phylogeographic studies. The tMRCAs and demographic analyses of the three relict species suggest that they began expanding at different times and experienced distinct survival histories. Both their similarities and differences may have been influenced by spatiotemporal changes in snow cover. In addition, their distinct survival histories, as inferred from their population genetic structures and diversity, suggest that life-history traits have also played an important role (Duminil et al. 2007). Overall, this study suggests that climatic oscillations during the Pleistocene led to extinctions followed by subsequent expansions of certain lineages. On the other hand, analysis using nuclear genomic regions with biparental inheritance may reveal more detailed changes in plants inhabiting snowy regions, and additional examples from other snow-dependent plants using nuclear DNA would further support this hypothesis (Masuda et al. 2024).

## Supplementary Information

Below is the link to the electronic supplementary material.

Supplementary file 3 (PDF 639 KB)
