# Supplementary material for: Reconstructing the survival history of relict herbs endemic to the heavy snowfall regions of Japan during the Pleistocene
Source: J Plant Res. 2026 Jun 22;139(5):731–43. doi: 10.1007/s10265-026-01727-y (PMC13588925; doi:10.1007/s10265-026-01727-y)
Supplement: Supplementary file 1 [file 10265_2026_1727_MOESM1_ESM.pdf]

Supplementary Information

Title:

Reconstructing the survival history of relict herbs endemic to the heavy snowfall regions of Japan during the Pleistocene

Journal:

Journal of Plant Research

Authors:

Watanabe Yoichi [1]*, Momoka Gozono [2], Hinata Koide [2], Hotaka Ishida [2], Koki Nagasawa [3], Kazutoshi Masuda [4], Shota Sakaguchi [5]

[1] Graduate School of Horticulture, Chiba University, Matsudo 648, Matsudo, Chiba 271-8510, Japan

[2] Faculty of Horticulture, Chiba University, Matsudo 648, Matsudo, Chiba 271-8510, Japan

[3] Research Center of Genetic Resources, National Agriculture and Food Research Organization, Kan-nondai, Tsukuba, 3058517, Japan

[4] Graduate School of Human and Environmental Studies, Kyoto University, Yoshida-nihonmatsu-cho, Sakyo, Kyoto 606-8501, Japan

[5] Graduate School of Agriculture, Kyoto University, Kitashirakawa-oiwake-cho, Sakyo, Kyoto 606-8502, Japan

* Correspondence: ashitaka3776@gmail.com

Table S1 Sampling location and genetic diversity estimates for *Glaucidium palmatum*, *Ranzania japonica* and *Paris japonica*

Species	Region	Locality	Latitude	Longitude	Altitude (m)	Snow depth (cm)	*N*	*h*	Haplotypes
Glaucidium palmatum									
	Hokkaido	Mt. Yubari, Yubari City, Hokkaido Pref., Hokkaido Isl.	43.11	142.22	1301	117	1	1	gH37(1)
		Mt. Kannon, Samani Town, Hokkaido Pref., Hokkaido Isl.	42.13	142.91	92	41	3	3	gH28(1), gH29(1), gH30(1)
		Heiwa-waterfall, Sapporo City, Hokkaido Pref., Hokkaido Isl.	43.06	141.22	255	146	4	2	gH9(2), gH10(2)
		Kannon-waterfall, Furubira Town, Hokkaido Pref., Hokkaido Isl.	43.23	140.59	89	152	3	2	gH18(2), gH19(1)
		Mt. Mekunnai, Rankoshi Town, Hokkaido Pref., Hokkaido Isl.	42.90	140.54	794	432	4	2	gH22(3), gH23(1)
		Utasai, Kuromatsunai Town, Hokkaido Pref., Hokkaido Isl.	42.65	140.32	46	128	5	3	gH10(3), gH33(1), gH34(1)
		Sasayama, Esashi Town, Hokkaido Pref., Hokkaido Isl.	41.86	140.20	302	48	4	2	gH3(3), gH4(1)
		Mt. Hakodate, Hakodate City, Hokkaido Pref., Hokkaido Isl.	41.75	140.71	200	28	4	1	gH12(4)
	northern Honshu	Mt. Kodake, Fujisato Town, Akita Pref., Honshu Isl.	40.42	140.21	720	185	4	2	gH15(3), gH16(1)
		Yunosawa, Senboku City, Akita Pref., Honshu Isl.	39.81	140.79	740	319	5	2	gH35(4), gH36(1)
		Ooshimizu-enchi, Yurihonjo City, Akita Pref., Honshu Isl.	39.10	140.11	840	394	4	1	gH27(4)
		Mt. Kanegasaki-komagatake, Kitakami City, Iwate Pref., Honshu Isl.	39.20	140.92	1020	160	4	2	gH13(3), gH14(1)
		Mt. Gassan, Nishikawa Town, Yamagata Pref., Honshu Isl.	38.54	140.01	1589	450	4	2	gH5(3), gH6(1)
		Mt. Torihara, Asahi Town, Yamagata Pref., Honshu Isl.	38.27	139.96	1363	324	2	2	gH31(1), gH32(1)
		Mt. Iide, Kitakata City, Fukushima Pref., Honshu Isl.	37.87	139.71	1700	268	2	2	gH20(1), gH21(1)

		Mt. Handa, Koori Town, Fukushima Pref., Honshu Isl.	37.88	140.50	440	31	4	2	gH7(3), gH8(1)
	central Honshu	Mt. Aizu-komagatake, Hinoemata Village, Fukushima Pref., Honshu Isl.	37.02	139.34	1850	213	4	2	gH1(1), gH2(3)
		Mt. Naeba, Yuzawa Town, Niigata Pref., Honshu Isl.	36.85	138.70	1876	234	3	2	gH2(2), gH11(1)
		Mt. Yoneyama, Kashiwazaki City, Niigata Pref., Honshu Isl.	37.30	138.49	729	85	4	1	gH2(4)
		Mt. Kurosawa, Myoko City, Niigata Pref., Honshu Isl.	36.89	138.08	1780	238	4	1	gH1(4)
		Mt. Kurohime, Itoigawa City, Niigata Pref., Honshu Isl.	36.98	137.79	1034	127	1	1	gH17(1)
		Nagashiri-sawa, Hakuba Village, Nagano Pref., Honshu Isl.	36.74	137.78	1608	184	3	2	gH24(2), gH25(1)
		Oh'gi-sawa, Ohmachi City, Nagano Pref., Honshu Isl.	36.56	137.71	1575	112	3	1	gH26(3)
Ranzania japonica									
	northern Honshu	Ajigasawa, Ajigasawa Town, Aomori Pref., Honshu Isl.	40.57	140.10	698	304	2	1	rH1(2)
		Yunosawa, Senboku City, Akita Pref., Honshu Isl.	39.81	140.79	740	319	2	1	rH1(2)
		Mt. Kanegasaki-komagatake, Kitakami City, Iwate Pref., Honshu Isl.	39.20	140.92	1020	160	5	1	rH1(5)
		Ooshimizu-enchi, Yurihonjo City, Akita Pref., Honshu Isl.	39.10	140.11	840	394	4	2	rH1(3), rH4(1)
		Oiri-sawa, Obanazawa City, Yamagata Pref., Honshu Isl.	38.52	140.57	601	281	3	1	rH1(3)
		Mt. Yudono, Tsuruoka City, Yamagata Pref., Honshu Isl.	38.52	139.96	880	397	4	4	rH5(1), rH6(1), rH7(1), rH8(1)
		Oishi-zawa, Oguni Town, Yamagata Pref., Honshu Isl.	37.96	139.80	436	199	2	1	rH1(2)
	central Honshu	Danyoshi-shindo, Hinoemata Village, Fukushima Pref., Honshu Isl.	36.96	139.26	1426	268	6	2	rH1(5), rH2(1)

		Sotonosawa, Tookamachi City, Niigata Pref., Honshu Isl.	36.94	138.71	929	222	4	1	rH1(4)
		Mt. Togakushi, Nagano City, Nagano Pref., Honshu Isl.	36.79	138.08	1475	176	5	1	rH1(5)
		Nagashiri-sawa, Hakuba Village, Nagano Pref., Honshu Isl.	36.74	137.78	1650	184	7	2	rH1(6), rH3(1)
		Sarukura, Hakuba Village, Nagano Pref., Honshu Isl.	36.74	137.81	1124	172	6	1	rH1(6)
Paris japonica									
	northern Honshu	Mt. Jinba, Hirosaki City, Aomori Pref., Honshu Isl.	40.48	140.34	900	148	4	4	pH24(1), pH25(1), pH26(1), pH27(1)
		Naganuma, Kazuno City, Akita Pref., Honshu Isl.	39.98	140.82	1090	311	4	4	pH30(1), pH51(1), pH52(1), pH53(1)
		Mt. Mokko, Senboku City, Akita Pref., Honshu Isl.	39.94	140.85	1447	301	3	3	pH38(1), pH39(1), pH40(1)
		Mt. Yakeishi, Oushu City, Iwate Pref., Honshu Isl.	39.17	140.81	1200	435	3	3	pH30(1), pH61(1), pH62(1)
		Mt. Kamuro, Yuzawa City, Akita Pref., Honshu Isl.	38.91	140.50	1260	372	4	4	pH23(1), pH28(1), pH29(1), pH30(1)
		Mt. Gassan, Nishikawa Town, Yamagata Pref., Honshu Isl.	38.54	140.00	1438	441	4	4	pH10(1), pH11(1), pH12(1), pH13(1)
		Mt. Nishi-Azuma, Yonezawa City, Yamagata Pref., Honshu Isl.	37.75	140.15	1880	154	3	2	pH30(1), pH7(2)
		Mt. Iide, Kitakata City, Fukushima Pref., Honshu Isl.	37.84	139.67	1900	251	3	3	pH35(1), pH36(1), pH37(1)
	central Honshu	Mt. Aizu-komagatake, Hinoemata Village, Fukushima Pref., Honshu Isl.	37.01	139.33	1700	220	5	5	pH3(1), pH20(1), pH21(1), pH22(1), pH23(1)
		Mt. Hotaka, Kawaba Village, Gunma Pref., Honshu Isl.	36.80	139.14	2050	145	2	2	pH18(1), pH19(1)
		Mt. Naeba, Sakae Village, Nagano Pref., Honshu Isl.	36.85	138.68	1750	232	2	2	pH43(1), pH44(1)

Mt. Kurosawa, Myoko City, Niigata Pref., Honshu Isl.	36.91	138.09	2110	217	7	6	pH9(1), pH23(2), pH31(1), pH32(1), pH33(1), pH34(1)
Nagashiri-sawa, Hakuba Village, Nagano Pref., Honshu Isl.	36.74	137.78	1633	184	7	7	pH41(1), pH42(1), pH45(1), pH46(1), pH47(1), pH48(1), pH49(1)
Oh'gi-sawa, Ohmachi City, Nagano Pref., Honshu Isl.	36.56	137.71	1575	112	6	6	pH54(1), pH55(1), pH56(1), pH57(1), pH58(1), pH59(1)
Mt. Chogatake, Azumino City, Nagano Pref., Honshu Isl.	36.29	137.73	2550	73	6	6	pH4(1), pH5(1), pH6(1), pH7(1), pH8(1), pH9(1)
Mt. Tateyama, Tateyama Town, Toyama Pref., Honshu Isl.	36.57	137.56	1994	181	1	1	pH60(1)
Amo, Hida City, Gifu Pref., Honshu Isl.	36.24	136.96	1495	147	2	2	pH1(1), pH2(1)
Mt. Hakusan, Hakusan City, Ishikawa Pref., Honshu Isl.	36.14	136.75	1913	248	4	4	pH14(1), pH15(1), pH16(1), pH17(1)

N, number of samples; *h*, number of haplotypes.

Snow depth, maximum snow depth of population location. Maximum snow depth values were extracted from Japan Meteorological Agency data with 30 arc-sec resolution in the latitudinal direction (https://www.data.jma.go.jp/obd/stats/etrn/view/atlas.html).

Table S2 Primer pairs used for each species

Region	Primer	*Glaucidium palmatum*	*Ranzania japonica*	*Paris japonica*
Z1	CPUP_1F_trnK-UUU<br>CPUP_1R_trnR-UCU	O	O	O
Z2	CPUP_2F_trnR-UCU<br>CPUP_2R_rpoC2	O	O	O
Z3	CPUP_3F_rpoC2<br>CPUP_3R_trnD-GUC	-	-	O
Z4	CPUP_4F_trnD-GUC<br>CPUP_4R_psaA	O	O	O
Z5	CPUP_5F_psaA<br>CPUP_5R_trnF-GAA	O	O	O
Z6	CPUP_6F_trnF-GAA<br>CPUP_6R_rbcL	O	O	O
Z7	CPUP_7F_rbcL<br>CPUP_7R_trnW-CCA	O	O	O
Z8	CPUP_8F_trnW-CCA<br>CPUP_8R_petD	O	O	O
Z9	CPUP_9F_petD<br>CPUP_9R_trnI-CAU	O	O	O
Z10	CPUP_10F_trnI-CAU<br>CPUP_10R_rps7	-	-	O
Z11	CPUP_11F_rps7<br>CPUP_11R_rrn4.5	O	O	O
Z12	CPUP_12F_rrn4.5<br>CPUP_12R_trnL-UAG	O	O	O
Z13	CPUP_13F_trnL-UAG<br>CPUP_13R_rps15	O	-	O
Z14	CPUP_14F_rps15<br>CPUP_14R_trnR-ACG	O	-	O
Z15	CPUP_15-1_1F_trnI-CAU<br>CPUP_15-1R_rps16	O	O	O
U4	rpoC2.520R<br>petN.3R	O	O	-
U5	trnC.GCA.47F<br>psaB.2170F	O	O	-
U11	rpl16.3R<br>ycf2.5100R	O	O	-
U12	ycf2.2300F<br>rps12.190F	O	O	-
U15	ndhI.194R<br>trnN.GUU.7R	-	O	-

Z1-Z15 (Zhang et al. 2016), U4-U15 (Uribe-Convers et al. 2014).

Reference:

Uribe-Convers S, Duke JR, Moore MJ, Tank DC (2014) A long PCR–based approach for DNA enrichment prior to next-generation sequencing for systematic studies. Appl Plant Sci 2: 1300063

Zhang T, Zeng CX, Yang JB, Li HT, Li DZ (2016) Fifteen novel universal primer pairs for sequencing whole chloroplast genomes and a primer pair for nuclear ribosomal DNAs. J Syst Evol 54: 219-227

Table S3 Sequences of outgroup species used

Species	outgroup species	Accession number
Glaucidium palmatum (Ranunculaceae + Berberidaceae)		
	Aconitum kusnezoffii	MK569468
	Adonis sutchuenensis	MK569470
	Anemone raddeana	MK569472
	Coptis chinensis	MK569483
	Eranthis stellate	MK569487
	Hydrastis canadensis	MK569495
	Ranzania japonica	MH423072
Ranzania japonica (Berberidaceae)		
	Achlys triphylla	MH423064
	Berberis amurensis	KM057374
	Berberis bealei	KF176554
	Caulophyllum robustum	MH423066
	Nandina domestica	DQ923117
	Podophyllum peltatum	MH423071
	Vancouveria hexandra	MH423073
Paris japonica (Melanthiaceae)		
	Chionographis japonica	KF951065
	Paris verticillata	MH796669
	Paris quadrifolia	KX784051
	Paris marmorata	KX784047
	Paris dunniana	KX784042
	Veratrum patulum	KF437397
	Xerophyllum tenax	KM078035
	Ypsilandra thibetica	MH796671

Table S4 Number of sites (bp) determined with no missing, and polymorphisms detected

	With outgroups			Without outgroups				
	number of sites	number of SNPs	Number of haplotypes*	number of sites	number of SNPs	Number of haplotypes	Number of shared haplotypes among populations	Number of singleton haplotypes
Glaucidium palmatum	65,091	2344	30 (7, 23)	115,061	244	37	3	21
Ranzania japonica	66,363	286	15 (7, 8)	113,592	15	8	1	7
Paris japonica	56,918	1347	61 (8, 53)	84,388	115	62	3	58

* Numbers of haplotypes for outgroups and the study species, respectively, are shown separately in parentheses.

SNP, single nucleotide polymorphism.

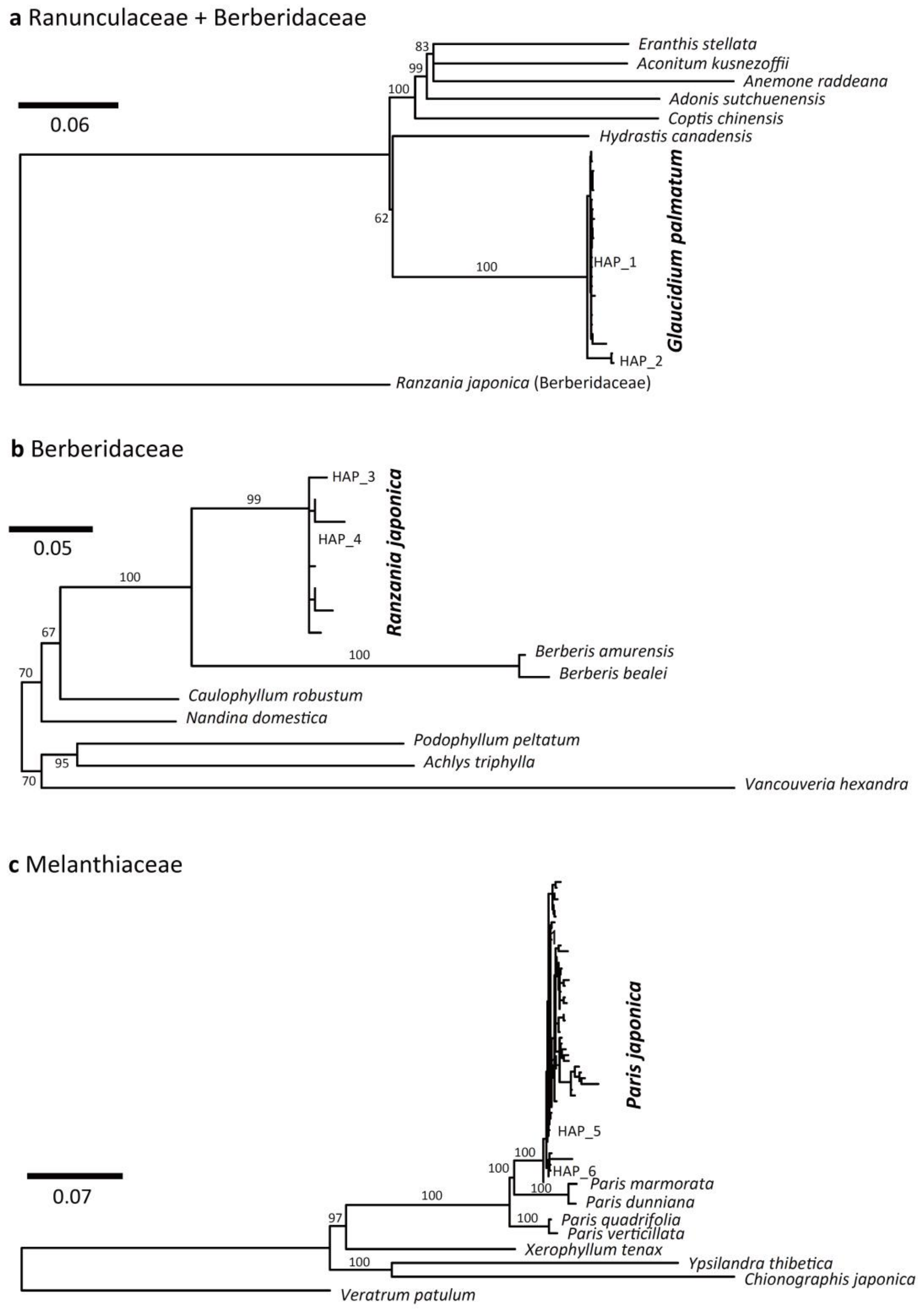

Fig. S1 Relationships between haplotypes from **a** Ranunculaceae including *Glaucidium palmatum* and Berberidaceae, **b** Berberidaceae including *Ranzania japonica*, and **c** Melanthiaceae including *Paris japonica*, estimated by a maximum likelihood method implemented in RAxML. Bootstrap probabilities are shown above nodes.

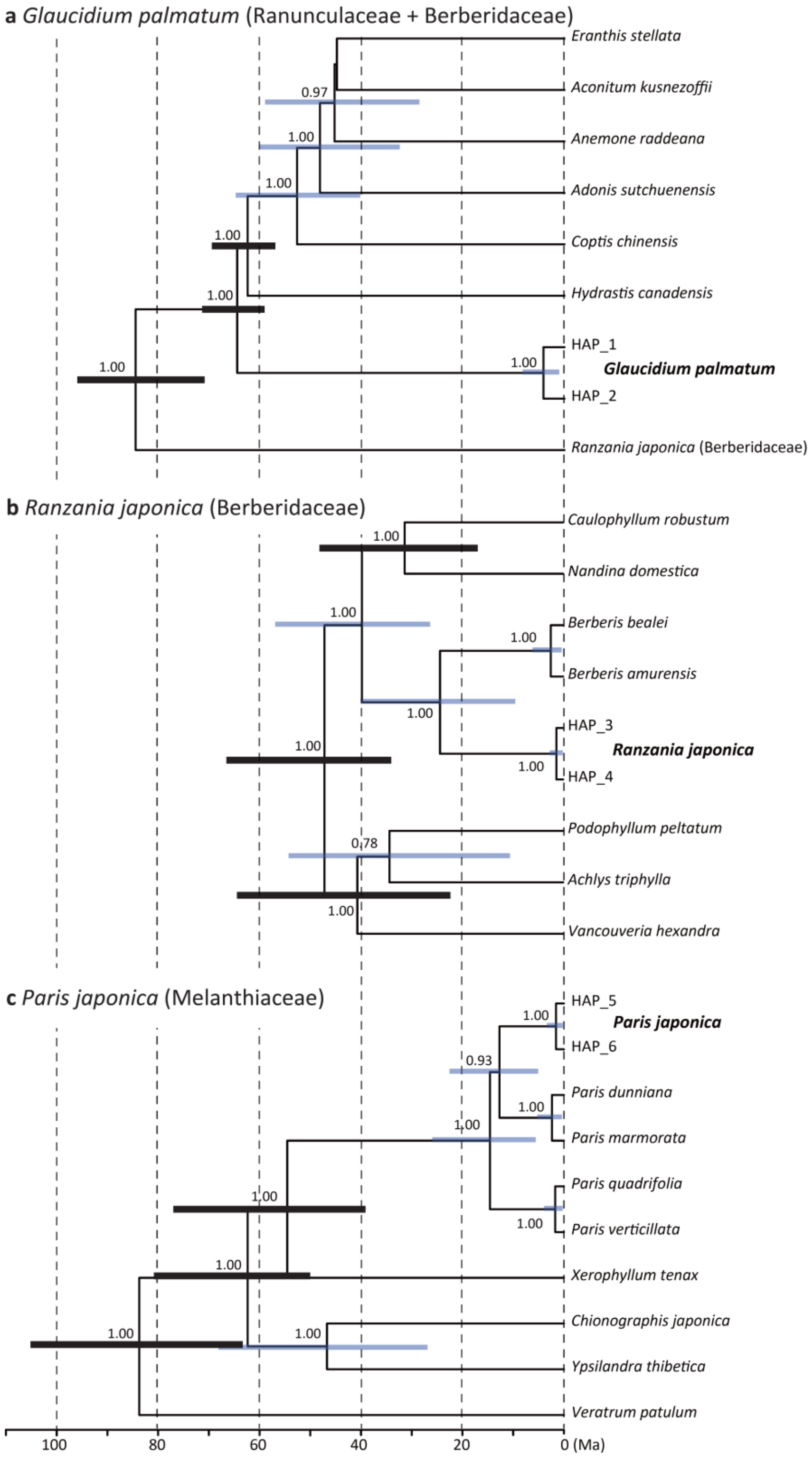

Fig. S2 Phylogenetic relationships and divergence time estimates for **a** Ranunculaceae and Berberidaceae, **b** Berberidaceae, and **c** Melanthiaceae. All posterior probabilities are shown above the nodes. Bars shown the 95% highest posterior density (95%HPD) of divergence time. Calibrated nodes are shown by black bars.

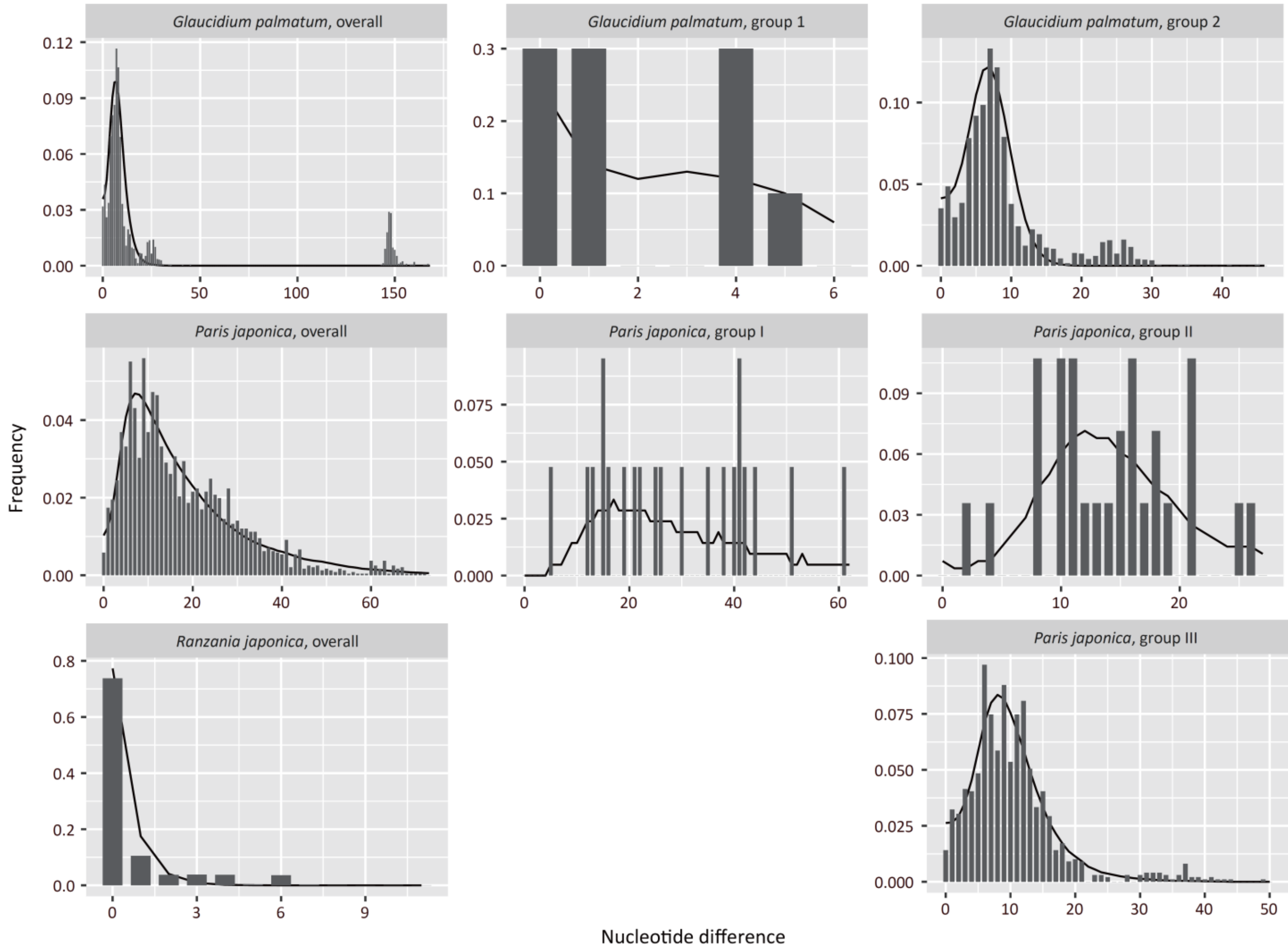

Fig. S3 The mismatch distribution for each study species and group. Bars show observed nucleotide differences between samples, and lines show the expected distribution under a sudden population expansion model.
